# Case Report: Clinical manifestations of uncommon monogenic disorders: revisiting activated phosphoinositide 3-kinase delta syndrome 2

**DOI:** 10.3389/fped.2025.1570600

**Published:** 2025-04-29

**Authors:** Oded Shamriz, Amarilla Mandola, Amos J. Simon, Atar Lev, Pierre Attal, Chen Nadler, Ortal Barel, Yulia Khavkin, Rachel Eisenberg, Raz Somech, Ori Toker

**Affiliations:** ^1^ Allergy and Clinical Immunology Unit, Department of Medicine, Hadassah Medical Organization, Faculty of Medicine, Hebrew University of Jerusalem, Jerusalem, Israel; ^2^ The Lautenberg Center for Immunology and Cancer Research, Faculty of Medicine, Institute of Medical Research Israel-Canada, Hebrew University of Jerusalem, Jerusalem, Israel; ^3^ Pediatric Department A and the Immunology Service, Jeffrey Modell Foundation Center, Tel-Hashomer Medical Center, Affiliated to the Sackler Faculty of Medicine, Edmond and Lily Safra Children’s Hospital, Tel Aviv University, Tel Aviv, Israel; ^4^ Sheba Cancer Research Center and Institute of Hematology, Sheba Medical Center, Tel Hashomer, Ramat Gan, Israel; ^5^ Department of Otolaryngology and Head and Neck Surgery, Shaare-Zedek Medical Center, Faculty of Medicine, Hebrew University of Jerusalem, Jerusalem, Israel; ^6^ Maxillofacial Imaging, Department of Oral Medicine, Sedation and Imaging, Hadassah Medical Center, Hebrew University of Jerusalem, Jerusalem, Israel; ^7^ The Genomic Unit, Sheba Cancer Research Center, Sheba Medical Center, Ramat Gan, Israel; ^8^ Sheba Medical Center, Wohl Institute of Translational Medicine, Ramat Gan, Israel; ^9^ Allergy and Clinical Immunology Unit, Shaare Zedek Medical Centre, Faculty of Medicine, Hebrew University of Jerusalem, Jerusalem, Israel

**Keywords:** APDS2, *PIK3R1*, inborn errors of immunity, combined immune deficiencies, primary immune deficiencies (PID)

## Abstract

**Aim:**

Pediatricians are trained to identify recurrent or unusual infections in children, prompting evaluation for inborn errors of immunity (IEI). Some monogenic IEI, however, may present atypically. This study describes our experience with children diagnosed with activated phosphoinositide 3-kinase delta syndrome (APDS2) including unusual presentations.

**Methods:**

A retrospective review was conducted on two children diagnosed with APDS2 at Shaare Zedek and Sheba Tel-Hashomer Medical Centers in Israel. Both patients underwent immune assessments, genetic testing, and treatment between 2019 and 2024.

**Results:**

Two patients, a 17-year-old female (P1) and a 7-year-old male (P2), were diagnosed with APDS2 after presenting with recurrent juvenile parotitis (P1) and severe lymphadenopathy (P2). Immunologic evaluation revealed hypogammaglobulinemia and combined immune deficiency. Genetic testing identified *PIK3R1* variants (c.1425 + 1G > T in P1 and c.1425 + 1G > C in P2). Both received intravenous immunoglobulins and prophylactic antibiotics. P2 was treated with rapamycin, leading to resolution of lymphadenopathy.

**Conclusion:**

This report highlights the clinical presentation of APDS2, a rare monogenic IEI in children, including the atypical manifestation of RJP and the common feature of lymphadenopathy. Pediatricians should stay vigilant for red flags of IEI during clinical evaluations, as early diagnosis and multidisciplinary care are crucial for effective management.

## 1 Introduction

Pediatricians are trained to recognize children with recurrent or unusual infections and refer them for evaluation of inborn errors of immunity (IEI). The well-known 10 warning signs of IEI primarily address infection frequency, severity, and pathogens involved (1).

Advances in next-generation sequencing and immunological methods have expanded the number of known IEI disorders to nearly 500, as classified by the International Union of Immunological Societies (IUIS) (2). This growing list includes primary immune regulatory disorders (PIRD), which present with immune dysregulation such as atopy, autoimmunity, and malignancy, making diagnosis more challenging.

Activated phosphoinositide 3-kinase delta (PI3Kδ) syndrome (APDS) is an autosomal dominant IEI (1). It is characterized by recurrent infections, as well as immune dysregulation including autoimmunity and lymphoproliferation (2). Pathogenesis involves the hyperactivation of PI3Kδ, a critical regulator of B and T-cell lymphocyte function. PI3Kδ is composed of two subunits: PIK3CD and PIK3R1. Pathogenic variants in these genes lead to the hyperactivity of PI3Kδ, resulting in the development of APDS 1 and 2, respectively. Hyper-activated PI3Kδ induces alterations in lymphocyte populations including B-cell lymphopenia, inverse ratio of CD4^+^ to CD8^+^ T cells, impaired T-cell effector functions, increased T-cell senescence and exhaustion and low percentages of naïve CD4^+^ and CD8^+^ T cells. Additionally, hypogammaglobulinemia and increased immunoglobulin (Ig)M levels are noted (2, 3).

APDS exemplifies an IEI with diverse and sometimes unexpected clinical manifestations. In this multicenter study, our objective was to examine patients diagnosed with APDS2, including those who displayed atypical symptoms.

## 2 Methods

### 2.1 The patients and study design

This is a multicenter retrospective study. We conducted a retrospective analysis of patients diagnosed with APDS2 (P1 and P2), who were treated at two medical centers in Israel: Shaare Zedek Medical Center in Jerusalem and Sheba Tel-Hashomer Medical Center in Ramat-Gan. The APDS2 patients underwent immune workup, genetic diagnosis, and were treated between 2019 and 2024.

### 2.2 Genetic diagnosis of *PIK3R1* variants

The patients were diagnosed by exome sequencing (ES). P1's genetic diagnosis was conducted at Hadassah Medical Center and P2's at Sheba Tel Hashomer Medical Center. Methodology for ES was previously described (4, 5).

### 2.3 Immune analysis

#### 2.3.1 Immune phenotyping

Peripheral blood mononuclear cells (PBMCs) were analyzed by flow cytometry (NAVIOS; Beckman Coulter, USA). Immunofluorescence staining included antibodies against CD3, CD4, CD8, CD19, and CD56 (Beckman Coulter, USA). Age-matched reference values for lymphocyte subsets and immunoglobulins were obtained from Schatorjé et al. (6) and Jolliff et al. (7).

#### 2.3.2 T-cell receptor Vβ repertoire analysis

T-cell receptor (TCR) Vβ repertoire analysis was performed via flow cytometry using the Beta Mark TCR Vβ Repertoire Kit (Beckman Coulter, USA) as per the manufacturer's guidelines.

#### 2.3.3 Quantification of T-cell receptor excision circles

T-Cell Receptor Excision Circles (TRECs) copy numbers were determined using qRT-PCR. A standard curve was constructed with a plasmid containing known TRECs copy numbers, and the quantification was performed based on cycle threshold values.

### 2.4 Ethical review of the study

This study was approved by the institutional review boards (IRB) of Shaare Zedek and Tel-Hashomer medical centers. Written informed consent was obtained from the individual AND/OR minor's legal guardian for the publication of any potentially identifiable images or data included in this article.

## 3 Results

### 3.1 Clinical manifestations of the APDS2 patients

**Figure 1:**
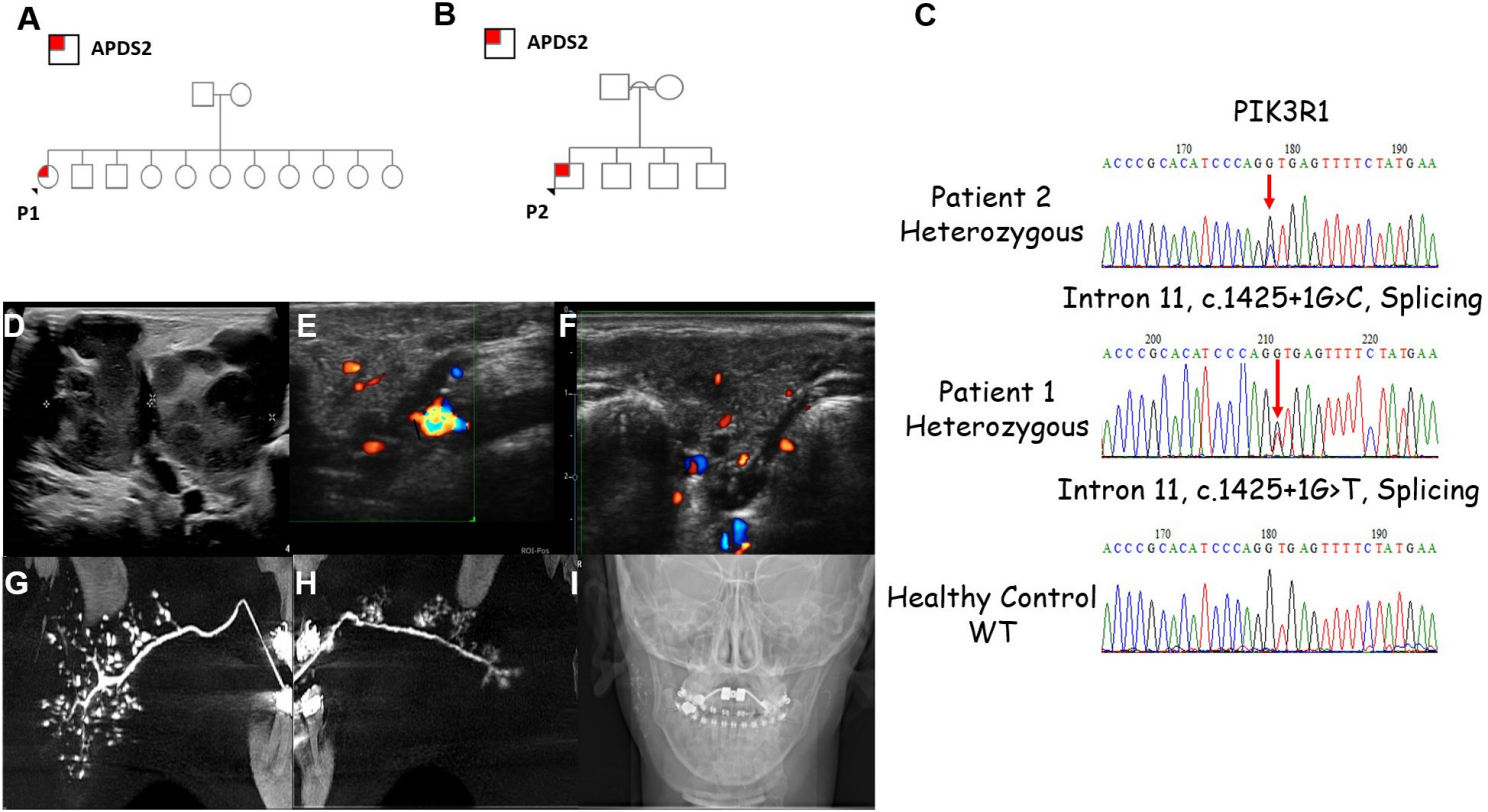
Clinical characteristics of the patients with APDS2. **(A,B)** Family pedigrees of P1 and P2, respectively. **(C)** Sanger sequencing demonstrating the *PIK3R1*variants found in P2 and P1. **(D)** Lymphadenopathy as demonstrated by ultrasound (US) scans of P2. Severely enlarged bilateral submandibular lymph nodes including small jugular lymph nodes can be seen. Non-homogenous pattern with some liquefication. The lengths of right and left submandibular lymph nodes are 4.36 and 2.1 cm, respectively. **(E,F)** US images of P1 of the right and left parotid glands showing hypoechoic round small (3–5 mm) regions, which may be compatible with sialectasis. **(G,H)** Bilateral sialo-cone beam computed tomography (sialo-CBCT) parotid gland image of P1 showing smooth main duct without sausaging (in left side mild dilatation in the distal 1 cm of the main duct) with few secondary ducts (ductopenia); **(G)** right showing numerous sialectasis, compatible with recurrent juvenile parotitis (RJP); **(H)** few clustered of sialectasis, without demonstrating the whole gland, compatible with RJP with secondary gland atrophy. **(I)** Scout image showing remaining contrast medium right main duct and sialectasis, left in main duct, thus demonstrating impaired bilateral gland activity.

Our search yielded two patients, whose clinical characteristics are summarized in Table 1. Patient 1 (P1) is a 17-year-old female who presented with xerostomia and xerophthalmia, as well bilateral swellings of the parotid glands for several months, more prominent on the left side. Her medical history included eczema, cutis marmorata, lymphadenopathy, recurrent non-purulent conjunctivitis without identified pathogens and recurrent otitis media and pneumonia since the age of one. P1 is the 10th out of 11 siblings to non-consanguineous parents with no family history of recurrent infections or IEI (Figure 1A). She had no neurodevelopmental delay. Physical examination showed hepato-splenomegaly and mild swelling of the left parotid region. Intraoral examination identified gingivitis and pus discharge from the left Stenson's duct upon milking of the left parotid gland. An ultrasound of the parotid area revealed numerous hyperechoic round small regions (Figures 1E,F). sialo-cone-beam computerized tomography (sialo-CBCT)of both parotid glands, following 5 days antibiotic course, demonstrated bilateral ductopenia, sialectasis, and impaired bilateral gland activity with atrophy on the left side, leading to a diagnosis of bilateral recurrent juvenile parotitis (RJP) with secondary atrophy and left parotid chronic obstructive sialadenitis (Figures 1G–I). Notably, anti-Ro/SSA and anti-La/SSB antibodies titers for Sjogren's syndrome were not completed. A total body CT scan revealed left cervical and inguinal lymphadenopathy, with lymph nodes measuring up to 15 mm. In addition, bronchiectasis was observed in the left lower lobe, along with infiltrates in the right lower lobe. The infectious workup included serum Epstein–Barr virus (EBV) PCR, which was negative on repeated sampling, and cytomegalovirus (CMV) PCR, which detected a viral load of 1,038 copies/ml (normal range: <800 copies/ml). However, no CMV prophylaxis was initiated. The cervical lymph node biopsy produced a small, slightly fragmented core of lymphoid tissue with a vaguely nodular architecture. Immunohistochemical staining was performed for LCA, CD3, CD20, PAX5, CD30, CD15, BCL-2, BCL-6, CD10, and CD21. The specimen exhibited interfollicular hyperplasia composed of small lymphocytes, immunoblasts, and scattered neutrophils, findings consistent with a reactive or potentially infectious process. *In situ* hybridization (ISH) for EBV-encoded RNA (EBER) was also performed and yielded negative results.

**Table 1:** Clinical characteristics of patients with clinical manifestations of APDS2.

Pt	Sex/Ethnicity	Ages on presentation/diagnosis (years)	*PIK3R1* gene variant (novel or reported, zygosity, pathogenicity prediction)	Consanguinity/Family history	Clinical Manifestations						Treatment	Outcome/Current age (years)
					Develop-mental	Infectious	Inflammatory	Facial dysmorphism	GI	Skin		
P1	F/J	1/2.5	c.1425 + 1G > T (Reported*, heterozygous, pathogenic)	No/No	Short stature	Recurrent sinopulmonary infections	Eczema; Juvenile recurrent parotitis; recurrent conjunctivitis; lymphadenopathy	None	Splenomegaly	Cutis marmurata	IVIG	CR of eczema, no infections/17
P2	M/A	2/7	c.1425 + 1G > C (Reported*, heterozygous, pathogenic)	Yes/No	Short stature	Recurrent sinopulmonary infections	Persistent relapsing noninfectious cervical lymphadenopathy with febrile episodes; Recurrent non-purulent tonsillitis	Small eyes; mild hypertelorism; short philtrum; low set ears	Hepatosplenomegaly	None	IVIG, prophylactic trimethoprim and sulfamethoxazole, rapamycin	CR of adenopathy, no infections, catch-up growth/7

* Gene variants of P1 and P2 were previously reported by Deau et al. (8) and Carrie et al. (9), respectively.

Pt, patient; M, male; F, female; A, Arab; J, Jewish; FTT, failure to thrive; PIK3R1, phosphoinositide-3-kinase regulatory subunit 1; GI, gastrointestinal; IVIG, intravenous immunoglobulins; CR, clinical resolution.

Patient 2 (P2) is a 7-year-old male, born at term to consanguineous Arab parents (Figure 1B). He was small for gestational age (SGA), weighing 1,400 grams due to placental insufficiency. He presented with persistent relapsing non-infectious cervical lymphadenopathy (Figure 1D). His medical history includes recurrent sino-pulmonary infections and non-purulent conjunctivitis since age two, along with recurrent tonsillitis, fever, and enlarged cervical lymph nodes since age three, without an infectious cause. P2 did not undergo neurodevelopmental evaluation, however he attends regular school and grade for his age. Physical examination revealed hepatosplenomegaly and facial dysmorphism, including a distinctive triangular face, a prominent forehead, micrognathia. microphthalmia, mild hypertelorism, short philtrum, and low-set ears. Further diagnostic workup consisted of a CT scan showing multiple enlarged lymph nodes in the neck and upper mediastinum, along with several small nodules in the lingula, left lower and right upper lobes. Additionally, chest CT also demonstrated very mild bronchiectasis of the right upper lobe, right lower lobe and the lingual.

Abdominal ultrasonography revealed a 12 cm homogenous spleen and a homogenous liver. Comprehensive infectious evaluation, including bacterial and mycobacterial blood cultures and EBV and CMV PCR and serology, was negative. Cervical lymph node excision and biopsy showed an inflammatory process with no microorganisms. Features such as a round necrotic area surrounded by cytotoxic T lymphocytes and histiocytes, paucity of neutrophils and plasma cells, occasional “crescent”-shaped histiocytes, and MPOX positivity raised a differential diagnosis of a granulomatous process or histiocytic necrotizing lymphadenitis. Due to the small biopsy size and lack of distinct lymph node structures, no definitive diagnosis could be made. There was a paucity of B-lymphocytes and no malignancy. Tests for CD23, CD21, CD30, CMV, IgM, IgA, and ISH-EBER were all negative.

Additionally, P2 underwent a bone marrow biopsy, which ruled out lymphoma and showed no granulomas, though some reactive changes were noted. Immunostaining revealed normal trilineage staining with no blasts. CD20 was negative, while CD79a showed a few small B lymphocytes and plasma cells in the interstitium, with a decrease or absence of B lineage precursors. CD3 staining identified small T lymphocytes, and the CD4:CD8 ratio was approximately 1:2–3, indicating an inverse ratio.

### 3.2 Genetic analysis confirms pathogenic variants in *PIK3R1*

**Figure 2:**
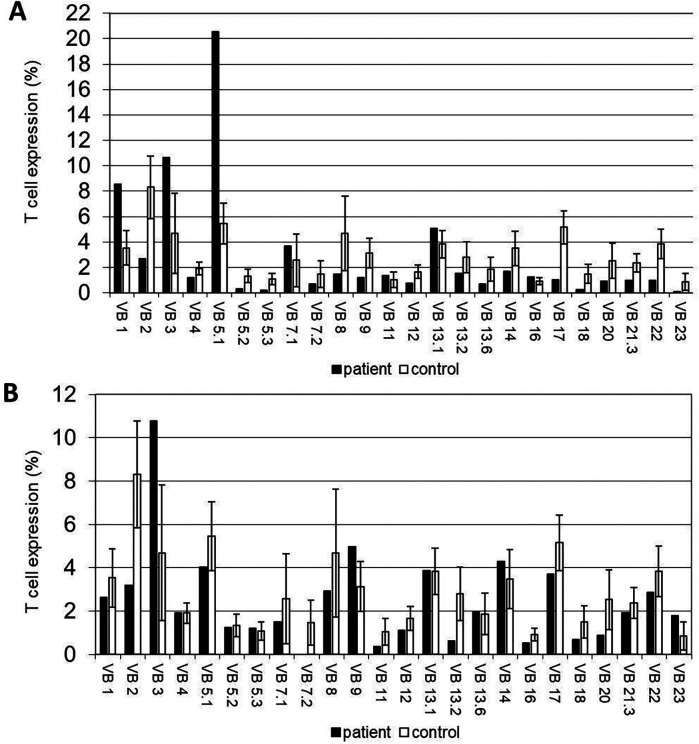
Immunological workup of the APDS2 patients. **(A,B)** T-cell receptor (TCR) V-β repertoires the APDS2 patients. **(A)** A restrictive pattern with the expansion of Vβ1, Vβ3 and Vβ5.1 clones and **(B)** a normal/polyclonal distribution are evident in P1 and P2, respectively.

Exome sequencing (ES) revealed variants in *PIK3R1*: c.1425 + 1G > T in P1 and c.1425 + 1G > C in P2 (Figure 2C). P1's variant was previously reported by Deau et al. (8), and P2's variant by Carrie et al. (9). Both variants were heterozygous and classified as pathogenic by various prediction software. Family segregation studies indicated that both P1's and P2's variants are *de novo*.

### 3.3 Immune investigation reveals a phenotype of combined immune deficiency

**Figure 3:**
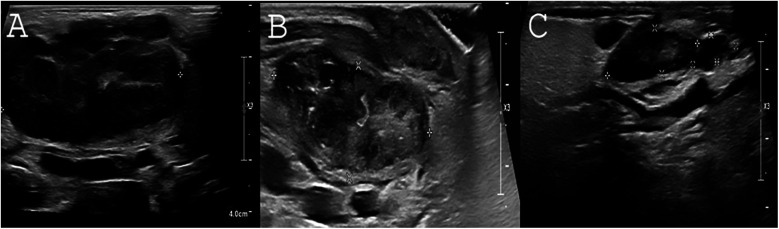
Response of lymphadenopathy to rapamycin treatment. **(A)** Severely enlarged bilateral submandibular lymph nodes as viewed by US upon P2's first examination. Length of right submandibular lymph node is measured 4.36 cm. **(B)** Two months following rapamycin treatment. A marked decrease of the right submandibular lymph node can be seen (3.6 × 1.1 × 1.4 cm) without abscess formation. **(C)** Following 1.5 years of rapamycin treatment, continuing decrease in the size of the right submandibular lymphadenopathy is noted (2.1 × 0.6 × 1 × 0.41 cm).

The immune workup of P1 and P2 is detailed in Table 2. Lymphocyte subset immune phenotyping revealed significant B-cell lymphopenia, with nearly absent B cells in both patients (11 cells/µl for P1 and 0 cells/µl for P2; age-matched reference ranges: 100–800 and 64–820 cells/µl, respectively). Both patients exhibited inverted CD4 to CD8 ratios and a significant decrease in the CD4^+^ T cell population. TRECs copy numbers were reduced in both patients (150 copies for P1 and 69 copies for P2; normal >400 copies). Additionally, P1's TCR V-β repertoire showed marked restriction with expansions in three clones, while repertoire of P2 was polyclonal (Figures 3A,B). Senescent T-cell or TEMRA-cell levels were not evaluated during the clinical workup and are therefore unavailable. Humoral investigation indicated hypogammaglobulinemia in both patients, with decreased levels of IgG, IgA, IgM, and IgE (546, 0, 7, and 0 mg/dl in P1 and 208, 0, 0, and 0 mg/dl in P2; age-matched reference ranges for IgG are 633–1,280 and 639–1,349 mg/dl for P1 and P2, respectively). Therefore, an immune phenotype of combined immune deficiency was evident in both P1 and P2.

**Table 2:** Immune workup of the patients diagnosed with APDS2.

Parameter			P1 (17 year-old)	P2 (7 year-old)	Age-matched normal range (5–10 years)*	Age-matched normal range (>16 years)*
Absolute leukocyte count (cells/µl)			5,900	12,770	4,500–13,500	4,500–11,000
Absolute lymphocyte count (cells/µl)			1,870	2,310	1,500–6,800	1,000–4,800
Absolute eosinophil count (cells/µl)			30	30	0–500	0–500
Immune phenotyping	T cells	CD3 (cells/µl)	1,501	2,486	770–4,000	780–3,000
		CD4 (cells/µl)	*265*	829	400–2,500	500–2,000
		CD8 (cells/µl)	1,144	1,437	200–1,700	200–1,200
		CD4:CD8	*0.2*	*0.58*	(1.5–2):1	(1.8–2.2):1
	NK cells	CD56 (cells/µl)	*691*	485	70–590	1,000–1,200
	B cells	CD19 (cells/µl)	*11*	*0*	100–800	64–820
TCR V-β repertoire			Restricted with 3 clones	Normal/polyclonal	-	-
TRECs (copies)			*150*	*69*	>400	>400
Serum total Ig		IgG (mg/dl)	*546*	*208*	633–1,280	639–1,349
		IgA (mg/dl)	*0*	*0*	33–202	70–312
		IgM (mg/dl)	*7*	*0*	48–207	40–230
		IgE (U/ml)	*0*	*0*	1.03–161.3	3–100
Specific IgG antibodies		HBV surface (MIU/ml)	273.45	NA	>15	>10
Autoantibodies (IgG)		ANA	Negative	Negative	1:160	1:160

* Age-matched reference ranges for lymphocyte subsets and immunoglobulins were determined according to Schatorjé et al. (6). and Jolliff et al. (7), respectively.

APDS2, activated phosphoinositide 3-kinase delta syndrome 2; NA, data is not available; NK, natural killer; Ig, immunoglobulins; HBV, hepatitis B virus; ANA, anti-nuclear antibody. In *Italics—*below normal range.

### 3.4 Treatment, clinical response to rapamycin and outcome

Both patients received intravenous immunoglobulins (IVIG) and prophylactic trimethoprim-sulfamethoxazole. For P1, the IVIG dose was adjusted based on trough IgG levels, clinical symptoms, and body weight. To monitor treatment effectiveness, IgG trough levels were measured monthly before IVIG administration. At 16.5 years of age, P2 weighed 38 kg and received a monthly IVIG dose of 15 g. However, she continued to experience recurrent parotitis and required frequent antibiotic treatments, with IgG trough levels of 581 mg/dl. After increasing the IVIG dose to 25 g per month—when her weight reached 44 kg—she showed clinical improvement. Her prophylactic regimen of trimethoprim-sulfamethoxazole consisted of a tablet containing 400 mg of sulfamethoxazole and 80 mg of trimethoprim, taken three times a week. P2 has been receiving IVIG at a dose of 0.5 g/kg per dose every four weeks, along with PCP prophylaxis using trimethoprim-sulfamethoxazole at 2.5 mg/kg per dose, twice a day, three times a week. Immunoglobulin trough levels are monitored at each IVIG administration and have remained stable.

Hematopoietic stem cell transplantation (HSCT) was offered to both patients. P1 and her parents declined the procedure. P2 underwent an initial assessment for HSCT; however, due to his stable clinical condition, transplantation was not pursued.

For immunomodulatory treatment, P2 was treated with rapamycin, initiated at a dose of 2 mg/m²/day and maintained at the same dosage. The target therapeutic levels were set at 5–15 ng/ml. Rapamycin levels were first monitored one week after initiation, along with a blood lipid profile and complete blood count (CBC). Given normal results, a follow-up blood draw was performed two weeks later. Subsequently, rapamycin levels, CBC, chemistry panel with lipids, and immunoglobulin levels were monitored at each pediatric daycare visit for IVIG administration. Additionally, EBV and CMV PCR screening was conducted every three months, though routine BK virus screening was not performed. Rapamycin treatment is ongoing, with no adverse events observed. Both patients are currently alive and well under treatment, with no further recurrent sinopulmonary infections. Before rapamycin treatment, P2 had severely enlarged bilateral submandibular lymph nodes, with the right submandibular lymph node measuring 4.36 cm. After 2 and 18 months of rapamycin treatment, the size of the right submandibular lymph node significantly decreased to 3.6 × 1.1 × 1.4 cm and 2.1 × 0.6 × 0.41 cm, respectively. No abscess formation was noted. The sonographic resolution of lymphadenopathy in P2 under rapamycin treatment is depicted in Figures 3A–C*.*

## 4 Discussion

In this study, we describe two patients diagnosed with APDS2. P1 exhibited RJP as an atypical manifestation of APDS2, while P2 presented with severe lymphadenopathy, which is not an uncommon feature of APDS2, occurring in approximately 89% of cases (10).

Recurrent juvenile parotitis consists of episodic and painful swelling of the parotid gland, either unilaterally or bilaterally. Interestingly, RJP is listed second only to mumps as the underlying cause of parotitis in children (11). The disease course is mostly self-limiting within 10 years. Associations of RJP with Sjögren's syndrome, hypogammaglobulinemia, selective IgG3 deficiency and IgA deficiency have all been previously reported (11). however, the etiology of this sialadenitis remains unknown and is currently regarded as multifactorial (12).

Salivary gland involvement in APDS is considered rare. In a cohort of 53 APDS patients, only 3 were reported to have sialadenitis, all with parotid gland abscesses (13). Additionally, RJP was not included among the symptoms attributed to APDS in the European Society of Immunodeficiencies (ESID)-APDS registry analysis of 170 patients (14, 15). Our report indicates that disorders of the immune system, previously reported in RJP patients, could in fact underlie undiagnosed APDS in some patients. Diagnosis of APDS through immune and genetic workup is currently more available and should be considered by the treating physician in the presence of RJP, specifically when encountering different warning signs for IEI, such as recurrent sinopulmonary infections.

The ESID-APDS registry analysis found APDS to have the highest rates of lymphoproliferation compared to the clinically similar autoimmune lymphoproliferative IEIs Cytotoxic T-lymphocyte associated protein 4 (CTLA-4) haploinsufficiency, Signal transducer and activator of transcription 3 (STAT3) gain-of-function and Nuclear Factor Kappa B Subunit 1(NFkB1) deficiency (15). Corresponding to these results, P2's most prominent manifestation was severe lymphoproliferation, although he also had a history of recurrent infections. Diagnosing IEI characterized by immune dysregulation and lymphoproliferation can be extremely difficult, especially with lymphoproliferation that can mimic lymphoma (2). Diagnostic clues in P2, besides a history of infections, included syndromic features and short stature, both known to be observed in APDS2 (14, 15). Short stature is more common in APDS2 than APDS1 and is believed to be induced by general factors, such as failure to thrive due to recurrent infections, as well as over-activation of the PI3Kδ –related pathway (2). Additionally, neurodevelopmental delay occurs in up to 31% of patients (10), and in some cases, a combination of clinical findings defines SHORT syndrome (16). Regarding SHORT syndrome, besides a short stature with a standard deviation score of −2.6, P2 had no hernia or joint hyperextensibility. He exhibited ocular depression (deep-set eyes) but did not have Rieger anomaly or teething delay. He had facial dysmorphism. There was no evidence of lipodystrophy, insulin resistance, hearing loss, or delayed speech and motor development. However, intrauterine growth restriction (IUGR) is present. Thus, in patients with APDS a multidisciplinary approach should be used in the diagnostic workup of these patients, with combined treatment from hematologists, immunologists, and infectious disease specialists.

In both P1 and P2, delayed diagnoses were noted, with a delay between symptom onset and diagnosis of 16 years for P1 and 5 years for P2. Diagnosing APDS poses significant challenges. Both APDS1 and APDS2 are exceptionally rare, occurring in only 1–2 patients per million (2). Furthermore, the clinical presentation of APDS varies widely, with heterogeneous severity and unpredictable outcomes ranging from asymptomatic cases to high mortality rates in early childhood (13). Additionally, establishing a correlation between genotype and phenotype in APDS is difficult, making it challenging to predict disease progression even among different patients with the same genetic variant. For instance, patients harboring the same *PIK3CD* gene variant, such as E1021K, may experience divergent disease courses (15). Consequently, diagnosing APDS often experiences delays from initial presentation (17).

Early diagnosis of APDS is essential for ensuring that patients receive prompt and appropriate care. Intravenous immunoglobulins should be promptly administered to manage the hypogammaglobulinemia often seen in these patients. Attention should also be given to short stature and developmental delays (2). Rapamycin, which targets mTOR, a downstream protein of PI3Kδ, has proven effective in reducing lymphoproliferation, as seen in patient P2 of our cohort. However, its effectiveness in treating the autoimmunity and inflammatory aspects of APDS is limited (2). Alternatively, leniolisib, a PI3Kδ inhibitor, provides targeted therapy with less toxicity than rapamycin. Leniolisib is currently approved for APDS 1 and 2. A phase III randomized clinical trial demonstrated that leniolisib significantly reduced lymphoproliferation and had a positive effect on the immune system, including an increase in B cell numbers (18).

In conclusion, our report highlights typical and atypical manifestations of APDS2, a rare monogenic disorder. Treating physicians should be aware and actively look for red flags for IEI, as well as diagnostic clues during examination and general workup. Prompt immune and genetic workups are needed to diagnose APDS patients early. We recommend follow-up of these patients by a multidisciplinary team.

## Data Availability

The original contributions presented in the study are included in the article/Supplementary Material, further inquiries can be directed to the corresponding author/s.
